# Attenuating dependence on structural data in computing protein energy landscapes

**DOI:** 10.1186/s12859-019-2822-5

**Published:** 2019-06-06

**Authors:** David Morris, Tatiana Maximova, Erion Plaku, Amarda Shehu

**Affiliations:** 1 Department of Computer Science, George Mason University, Fairfax, 22030 VA USA; 2 Department of Electrical Engineering and Computer Science, The Catholic University of America, Washington, 20064 D.C. USA; 3 Department of Bioengineering, George Mason University, Fairfax, 22030 VA USA; 4 School of Systems Biology, George Mason University, Manassas, 20110 VA USA

**Keywords:** Protein energy landscape, Structural dynamics, Stochastic optimization

## Abstract

**Background:**

Nearly all cellular processes involve proteins structurally rearranging to accommodate molecular partners. The energy landscape underscores the inherent nature of proteins as dynamic molecules interconverting between structures with varying energies. In principle, reconstructing a protein’s energy landscape holds the key to characterizing the structural dynamics and its regulation of protein function. In practice, the disparate spatio-temporal scales spanned by the slow dynamics challenge both wet and dry laboratories. However, the growing number of deposited structures for proteins central to human biology presents an opportunity to infer the relevant dynamics via exploitation of the information encoded in such structures about equilibrium dynamics.

**Results:**

Recent computational efforts using extrinsic modes of motion as variables have successfully reconstructed detailed energy landscapes of several medium-size proteins. Here we investigate the extent to which one can reconstruct the energy landscape of a protein in the absence of sufficient, wet-laboratory structural data. We do so by integrating intrinsic modes of motion extracted off a single structure in a stochastic optimization framework that supports the plug-and-play of different variable selection strategies. We demonstrate that, while knowledge of more wet-laboratory structures yields better-reconstructed landscapes, precious information can be obtained even when only one structural model is available.

**Conclusions:**

The presented work shows that it is possible to reconstruct the energy landscape of a protein with reasonable detail and accuracy even when the structural information about the protein is limited to one structure. By attenuating the dependence on structural data of methods designed to compute protein energy landscapes, the work opens up interesting venues of research on structure-based inference of dynamics. Of particular interest are directions of research that will extend such inference to proteins with no experimentally-characterized structures.

## Background

Wet and dry laboratories have demonstrated that proteins switch between three-dimensional (3d) structures to accommodate different molecular partners in different cellular processes [1]. In particular, the structural rearrangements that a protein molecule undergoes under physiological conditions (at equilibrium) are both fast (and small) and slow (and large). Slow rearrangements occur on the nanosecond-to-millisecond time scale and allow a protein to access different functionally-relevant substates (often several Å apart). In the energy landscape that organizes the vast space of structures available to a protein by potential energies, slow structural rearrangements constitute paths that connect energy basins corresponding to different thermodynamically-stable and semi-stable structural states [2].

Characterizing the equilibrium structural dynamics of a protein is key to elucidating how protein structure modulates protein function [3]. Due to the diffusion time scales involved, it is not possible to probe all stable and semi-stable structural states or to reveal the detailed structure-by-structure rearrangements a protein uses to diffuse among such states in the wet laboratory. In principle, these issues can be addressed via a detailed characterization of the energy landscape in silico [2]. In practice, due to the disparate spatio-temporal scales involved, neither wet nor dry laboratories can reconstruct the energy landscape of any protein of interest [4]. Nonetheless, the challenges continue to spur computational research [3].

Two main challenges have been recognized with computational reconstructions of protein energy landscapes (and, more generally, biomolecular energy landscapes) [5]. The first relates to the high dimensionality of the structure space, which limits the sampling capability of a computational method. The second relates to inherent inaccuracies in molecular mechanics-based energy functions that evaluate atomic interactions in a structure and is known as the local minima (or ruggedness) issue.

While it remains challenging to reconstruct the energy landscape of a medium-size protein (100−300 amino acids long) that utilizes slow structural rearrangements to access different functionally-relevant substates, progress has been made in silico. This has been due to the fundamental realization that limited sampling capability is principally a variable selection issue [6]. Therefore, the sampling capability of a computational method can be enhanced via careful selection of the variables constituting the search space.

Recent efforts in silico have demonstrated that insight on variables underlying the slow dynamics of a protein is key to defining both a low-dimensional search space amenable to exploration and effective variation operators to obtain samples (new structures) under the umbrella of stochastic optimization [7–13]. In particular, proteins at the center of proteinopathies (such as many human cancers and neurological disorders), are avidly studied by many wet laboratories that report on stable and semi-stable states of healthy and diseased variants. The growing number of structures on such proteins presents an opportunity to make inferences on equilibrium structural dynamics. Recent successful algorithms leverage the growing number of structures deposited in public databases for healthy/wildtype (WT) and diseased/mutated forms of a protein. They extract the *extrinsic modes of motion* via Principal Component Analysis (PCA) of atomic displacements compiled from known structures of a protein. The extracted principal components (PCs) are utilized as variables/axes of the variable space. The space is then explored one sample at a time via iterative applications of selection (to select an existing sample) and variation (to obtain a new one) operators [11] under the umbrella of stochastic optimization.

This line of work has revealed precious insights on known and novel functionally-relevant states, the rearrangements between states, and the mechanisms via which mutations alter dynamics to cause dysfunction [7, 9, 13, 14]. However, the demand on sufficient prior structure data to define relevant variables limits broader applicability to proteins that are not as well studied in wet laboratories.

The key issue addressed in this paper is whether it is possible and to what extent one can reconstruct the energy landscape of a protein in the absence of sufficient, experimentally-available structural data. A complementary line of work in characterizing the slow dynamics of proteins presents an opportunity. Since the late 90s, normal mode (NM) analysis (NMA) has been established as an expedient technique via which to extract the *intrinsic modes of motion* (NMs) from a single structure [15, 16]. The low-frequency eigenvectors (slow modes) have been utilized to connect two structures (e.g., open/unbound and closed/bound) of a protein in algorithms seeking to elucidate a specific structural rearrangement between two known structures [17–20].

Here, we assess the extent to which the slow modes allow reconstructing the energy landscape of a protein (effectively, obtaining many structures out of one). We utilize a stochastic optimization framework, SoPriM [7], which allows plugging different variables of interest. While in prior work we have assessed the effectiveness of PCs as variables, here we assess the employment of the slow (NMA-extracted) modes for reconstruction of a protein energy landscape. We refer to the former algorithmic realization as SoPriM-PCA [7] and to the latter one, described and evaluated in this paper, as SoPriM-NMA. The objective is to assess in detail and in a controlled environment (on a protein that has been well studied by us and others) the landscape reconstructed when exploiting the dynamics encoded in only one structure (of the protein under investigation) versus the landscape that can be reconstructed when exploiting the dynamics encoded in a set of structures (caught for various forms of the protein under investigation).

We describe the proposed SoPriM-NMA in the “Methods” section, after summarizing the main algorithmic components of SoPriM (and SoPriM-PCA). We present a detailed evaluation in the “Results” section and conclude the paper with a summary and discussion of future directions of work.

## Methods

### SoPriM

The input to SoPriM is a set *Ω*_*S*_ of known structures of a protein and a matrix *U*_3*k*×3*k*_ encoding the variable space (each column encodes an axis, and *k* encodes the number of amino acids in the protein under investigation); *Ω*_*S*_ contains many structures, as in SoPriM-PCA, or a single structure, as in SoPriM-NMA. The structure(s) in *Ω*_*S*_ are projected onto the employed axes to obtain an initial population *Ω*_*C*_ of conformations, with each conformation being a point in the selected variable space. *Ω*_*C*_ initializes the desired population $\mathcal {C}$ of conformations. The SoPriM framework adds onto $\mathcal {C}$ via iterative application of a selection and a variation operator for a user-defined number of iterations (with iterations corresponding to the desired size of $\mathcal {C}$).

At every iteration, the selection operator selects a conformation from $\mathcal {C}$. The selection penalizes selecting conformations from over-populated or high-energy regions per a defined weighting function (over conformations and cells of a grid over two selected variables, as detailed in Ref. [7]). The selected conformation is then subjected to a variation operator that utilizes the variable axes (described below for the two different realizations SoPriM-PCA and SoPriM-NMA). Prior to adding a conformation resulting from an application of the variation operator to $\mathcal {C}$, the conformation is transformed into an all-atom structure. The transformation occurs over various scales. First, the conformation is converted to a CA trace (CA atoms), then to a backbone trace, then side chains are packed, and finally the resulting all-atom structure is minimized via the sander protocol with the Amber ff14SB force field. Details of this transformation protocol are available in Ref. [7]. The resulting structure is projected back into the variable axes to obtain the improved conformation for addition to the growing population $\mathcal {C}$.

### SoPriM-PCA

The selected variables are PCs; *U*_3*k*×3*k*_ is the set of eigenvectors obtained from a matrix *A* prepared as follows: Structures for the sequence under investigation (and variants no more than 3 mutations different) are collected from the PDB. The CA atoms are extracted from the *n* structures and stored in a matrix *A*_3*k*×*n*_ (we refer to a chain of CA atoms as a trace), and an average trace is computed. *A* is centered (by subtracting the average trace from each column of *A*) so that it encodes internal structural fluctuations rather than rigid-body motions in 3d. A singular value decomposition yields $1/\sqrt {n-1} \cdot A = U \cdot \Sigma \cdot V^{T}$. While further details can be found in Ref. [12], in summary, *U*_:,*i*_ contains the coordinates of PC _*i*_, and the singular values *Σ*_*ii*_ are square roots of eigenvalues *e*_*i*_ that measure the variance of the data (traces) when projected onto PC _*i*_. The order of the PCs in *U* is from high-to-low corresponding eigenvalues. A cumulative variance analysis allows selecting the top *m* PCs that cumulatively capture a threshold of structural variance (typically, 80%) as coordinate/variable axes. For many proteins with multiple functional states, even the top two PCs capture more than 50% of the variance.

Given *C* as a point in the space of the top *m* PCs, the variation operator computes a new conformation *C*_*new*_=*C* + *g*, where *g*=〈*g*_1_…*g*_*m*_〉 is a “global motion vector” that specifies displacements along each PC; *g*_*i*_=*s*_*i*_·*δ*_*i*_, where *s*_*i*_ is sampled uniformly at random in {−1,+1},*δ*_1_ is a user-defined parameter, and *δ*_*i*_=*δ*_1_·*e*_*i*_/*e*_1_ (for each *i*>1) to ensure that displacements are proportionate with the variations captured by each PC.

### SoPriM-NMA

In this setting, the NMs extracted from an NMA off a single structure are selected as variables. The reader is directed to seminal work in [15] for background and foundations of NMA in statistical mechanics. In practice, we employ the utilities in Bio3D [21] to extract the matrix *U*_3*k*×3*k*_ of the NMs off a single structure. Unlike PCA, the first 6 NMs capture rigid-body motions, so we discard them. From now on, NM7 through *NM*_3*k*−6_ are of interest for variable selection, and they are ordered by their associated frequencies (low to high, with low corresponding to slow modes). Let us renumber and refer to these frequency-ordered NMs of interest as NM_1_ through NM _*d*_ (*d*=3*k*−6). Prior to plugging them into SoPriM to obtain SoPriM-NMA, two questions need answering: (i) what *m*<<*d* to select as axes of the space; and (ii) how to utilize the selected *m* NMs to compute the global motion vector used by the variation operator. The first can be addressed by balancing between low dimensionality of the variable space and accurate reconstruction of known structures.

**Fig. 1:**
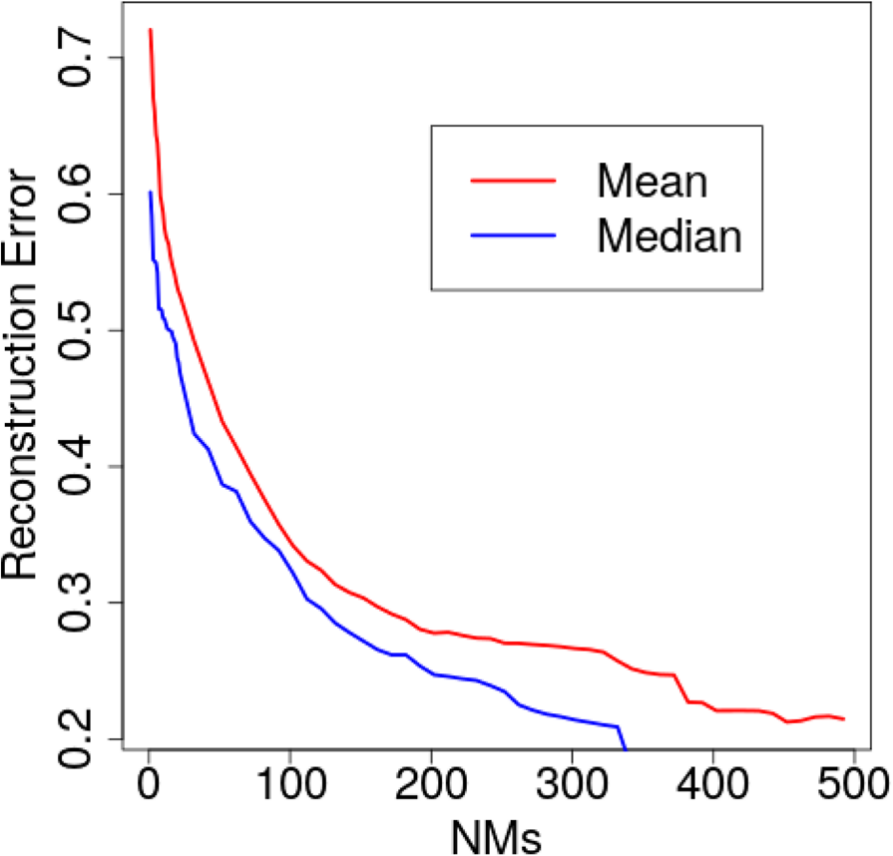
Loss Analysis: Mean and median lrmsds estimate the reconstruction error when using *i*≤*d* frequency-ordered NMs to recover CA traces of experimentally-known structures

Suppose that many structures are available for a protein of interest (as is the case for an enzyme employed here for this analysis), even though the NMs are extracted off a single selected structure. The CA traces of all structures are projected onto NM_1_, …, NM _*d*_ to obtain a corresponding *d*-dimensional point/conformation *C* for each trace. For a given *i*∈[*d*], for each of the conformations *C*, we can drop the other *d*−*i* coordinates (thus arbitrarily reducing the dimensionality of the space) to obtain a “reduced” conformation *C*_*i*_. For instance, if *i*=1,*C*_1_ contains only 1 coordinate (along NM_1_ in a 1-dim variable space); if *i*=*d*, all coordinates are retained. The transformation operation described above then allows reconstructing a CA trace from a conformation *C*_*i*_, and the least root-mean-squared-deviation (lrmsd) [22] between the reconstructed and the original trace can be recorded (for each of the structures). The mean and median lrmds can then be reported for a given value of *i*, as Fig. 1 does over known structures of the H-Ras enzyme, as *i* varies from 1 to *d* on the x axis.

Figure 1 shows that, as expected, the more NMs used, the lower the reconstruction error. This analysis also shows that the reconstruction error is less than 0.6Å even when less than 10 NMs are employed as variable axes, supporting studies showing that relatively few, low-frequency NMs can identify the direction of global motions required to achieve state-to-state transitions [19]. Such an analysis can be employed to select *m* <<*d* NMs as variables if many structures of a protein are available. When this is not the case, there is no general non-parametric rule for an optimal value for *m* besides the rule of thumb to keep the dimensionality low. In “Results” section we analyze in greater detail the relationship between NMs and PCs, focusing on a well-studied protein, H-Ras, and select *m* to be the same value whether employing PCs or NMs as variable axes.

#### Global motion vector

The global motion vector *g* is adapted from Ref. [19]: $g = \delta \cdot \sqrt {2/m} \cdot {\sum \nolimits }_{i=1}^{m} \frac {s_{i} \text {NM}_{i}}{f_{i}}$, where *δ* is a user-defined parameter, *s*_*i*_ is a sign sampled uniformly at random in {−1,+1} for each NM _*i*_ (so that displacements can be defined in the positive or negative direction along the principal axis of motion represented by an NM), and the scaling $\frac {1}{f_{i}}$ is so as to achieve a greater magnitude of displacement along lower-frequency NMs than along the higher-frequency modes under the same fixed energy (with frequencies corresponding to singular values of associated eigenvectors/NMs). This equation is based on the principle that displacements in the direction of each NM must produce a constant-valued energy when averaged over the resulting path, and the reader is directed to Ref. [19] for the underlying theory and derivation.

### Implementation details and experimental setup

A detailed analysis is conducted on a well-studied, 166-amino acid long enzyme, H-Ras, that populates various states. SoPriM-PCA utilizes 87 structures collected from the PDB for H-Ras WT and other variants. Three production runs are used to compute 45,000 structures (*δ*∈{1,2,3}). A detailed analysis in prior work shows these step sizes to balance between exploration and exploitation. SoPriM-NMA utilizes the NMs extracted from a single structure, instead. Two setups are considered, NMs extracted from the Amber ff14SB-minimized structure corresponding to H-Ras PDB entry 1QRA (a representative of the H-Ras GDP-bound/off state) and to H-Ras PDB entry 4Q21 (a representative of the GTP-bound/on state). Three production runs are employed under each setting to compute 45,000 structures (using *δ*∈{0.25,0.5,0.75}; an analysis on optimal values of *δ* is not shown here in the interest of space).

## Results

### Comparison of intrinsic to extrinsic motions

**Fig. 2:**
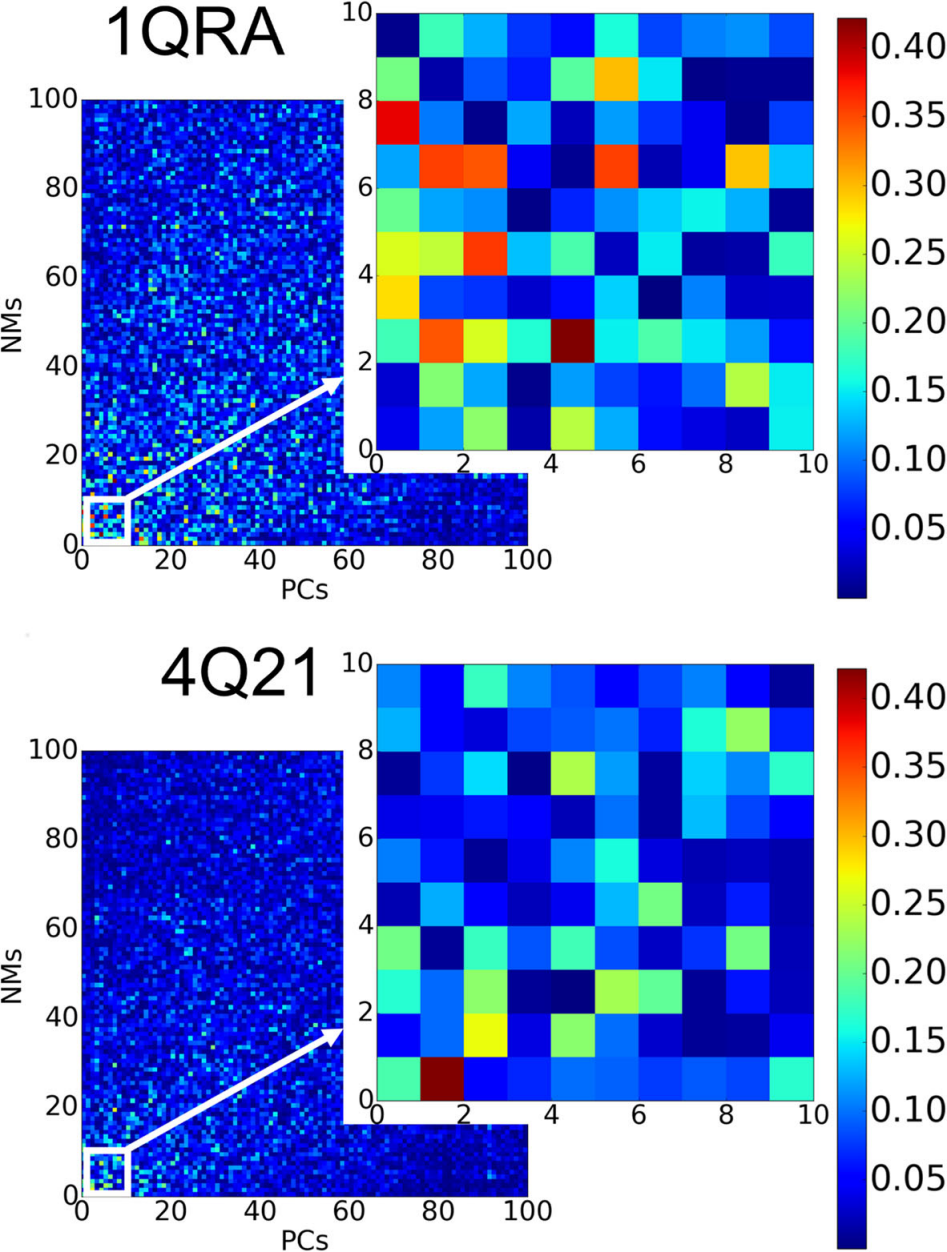
Comparison of NMs to PCs: Dot products are computed and shown color-coded between each of the top 100 PCs and 100 NMs (derived from 1QRA on the top panel and from 4Q21 on the bottom panel). The heatmap corresponding to the top 10 PCs and NMs is zoomed in. Low-to-high pairwise vector similarity is conveyed via a blue-to-red color scheme

PCs are compared directly to NMs via dot-products *NM*_*i*_·*PC*_*j*_ with *i,j* in [3*k*] (*k* being the number of CA atoms). Absolute values are used to color-code a heatmap. Figure 2 is limited to the top 100 PCs and top 100 NMs for ease of visualization; the PCs are ordered by their eigenvalues (high to low), and the NMs are ordered by their frequencies (low to high). The highest-similarity pairs are found among the top ten PCs and top ten NMs, as zoomed in on the right of the top and bottom panels of Fig. 2. Two setups are considered. The top panel of Fig. 2 analyzes the NMs derived from the (Amber ff14SB-minimized) off state representative structure (PDB id 1QRA). The bottom panel of Fig. 2 analyzes the NMS derived from the (Amber ff14SB-minimized) on state representative structure (PDB id 4Q21).

**Fig. 3:**
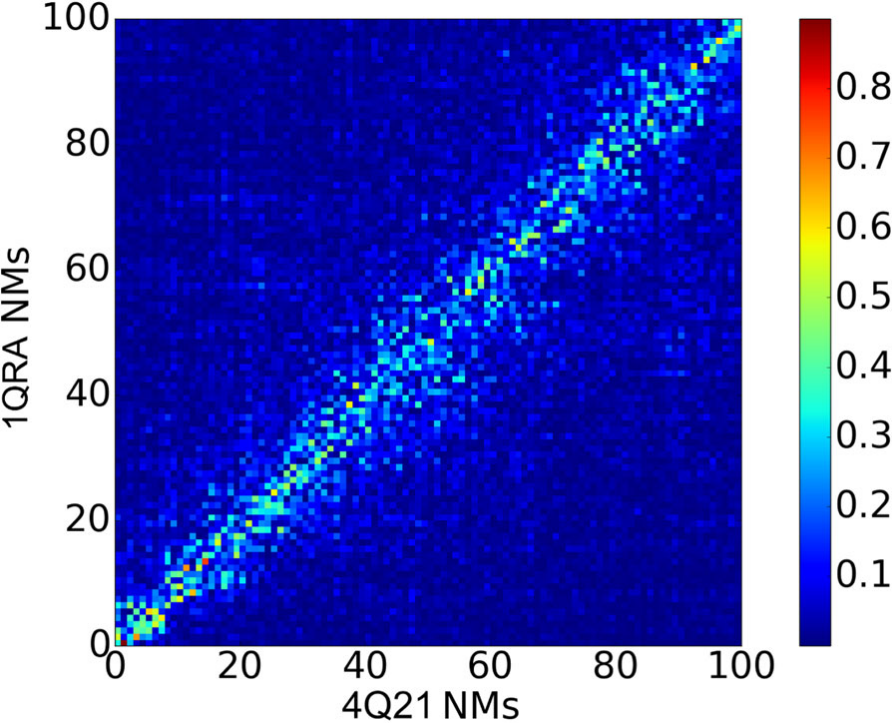
Comparison of NMs: Dot products are computed and shown color-coded between each of the top 100 NMs derived from 1QRA (x axis) and the top 100 NMs derived from 4Q21 (y axis). Low-to-high pairwise vector similarity is conveyed via a blue-to-red color scheme

Figure 2 shows that each of the top ten PCs, which capture more than 80% of the structural variance among known structures of H-Ras, is covered by at least one of the top ten NMs in each setting. In particular, PC1 and PC2 (which cumulatively capture more than 50% of the variance) are best captured by 1QRA-derived NM8 and NM7, respectively, and 4Q21-derived NM4 and NM1, respectively. These results support studies showing that highest-variance PCs correspond better to low-frequency NMs derived from closed (such as 4Q21) than open structures (1QRA). In addition, there is strong correspondence between the NMs derived from 1QRA and those derived from 4Q21, as shown in Fig. 3.

**Fig. 4:**
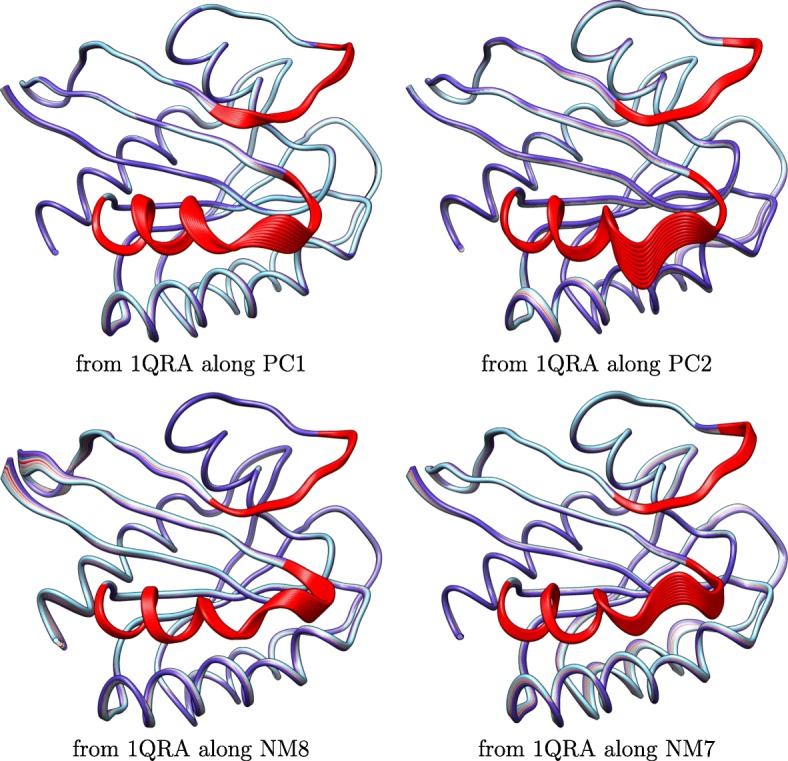
Visualization of Deformations of GDP-bound Structure: A few structures are generated by deforming the GDP-bound (off) representative structure (PDB id 1QRA) along a principal component or a normal mode. The structures are superimposed over the GDP-bound representative structure. The switch I and II functional regions are highlighted in red

**Fig. 5:**
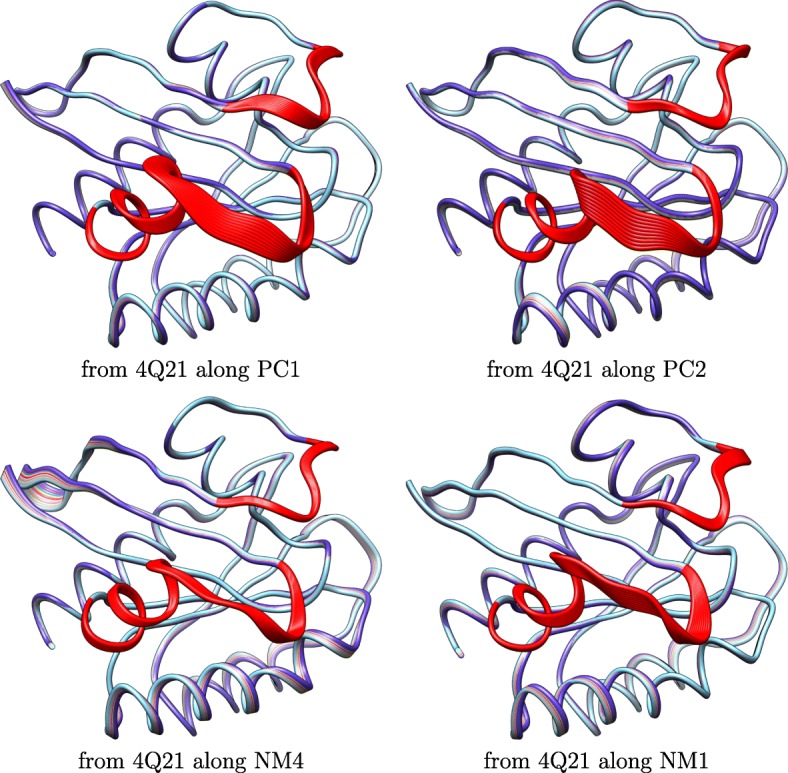
Visualization of Deformations of GTP-bound Structure: A few structures are generated by deforming the GTP-bound (on) representative structure (PDB id 4Q21) along a principal component or a normal mode. The structures are superimposed over the GTP-bound representative structure. The switch I and II functional regions are highlighted in red

The PCs-NMs correspondence is further visualized by drawing structures obtained along a selected axis (PC or NM). Instead of adding all the (properly-scaled) PCs or NMs in the global motion vector, only one PC or NM is selected over and over to produce 10 conformations at *δ*·*i* units away along the selected axis, with *i*∈[10] and using either the Amber ff14SB-minimized structure corresponding to PDB entry 1QRA or that to PDB entry 4Q21 as the selected start structure. The transformation summarized in Methods is utilized to obtain all-atom structures. Figure 4 shows 10 structures obtained by accumulating structural variations captured by PC1 or PC2 starting from 1QRA and 10 structures obtained by accumulating structural variations captured by NM8 or NM7 starting from 1QRA. Figure 5 shows 10 structures obtained by accumulating structural variations captured by PC1 or PC2 starting from 4Q21 and 10 structures obtained by accumulating structural variations captured by NM4 or NM1 starting from 4Q21. Figures 4 and 5 visually support the comparison related in Fig. 2 that NMs encode similar spatial displacements and affect the same functional regions, switch I and II (highlighted in red), of H-Ras.

These results suggest that one structure encodes similar information on the slow dynamics to what can be extracted when one has access to many known structures. While the top ten NMs contain the slow dynamics of interest, the first few (slowest) modes are more likely to capture this dynamics if extracted off a closed structure.

**Fig. 6:**
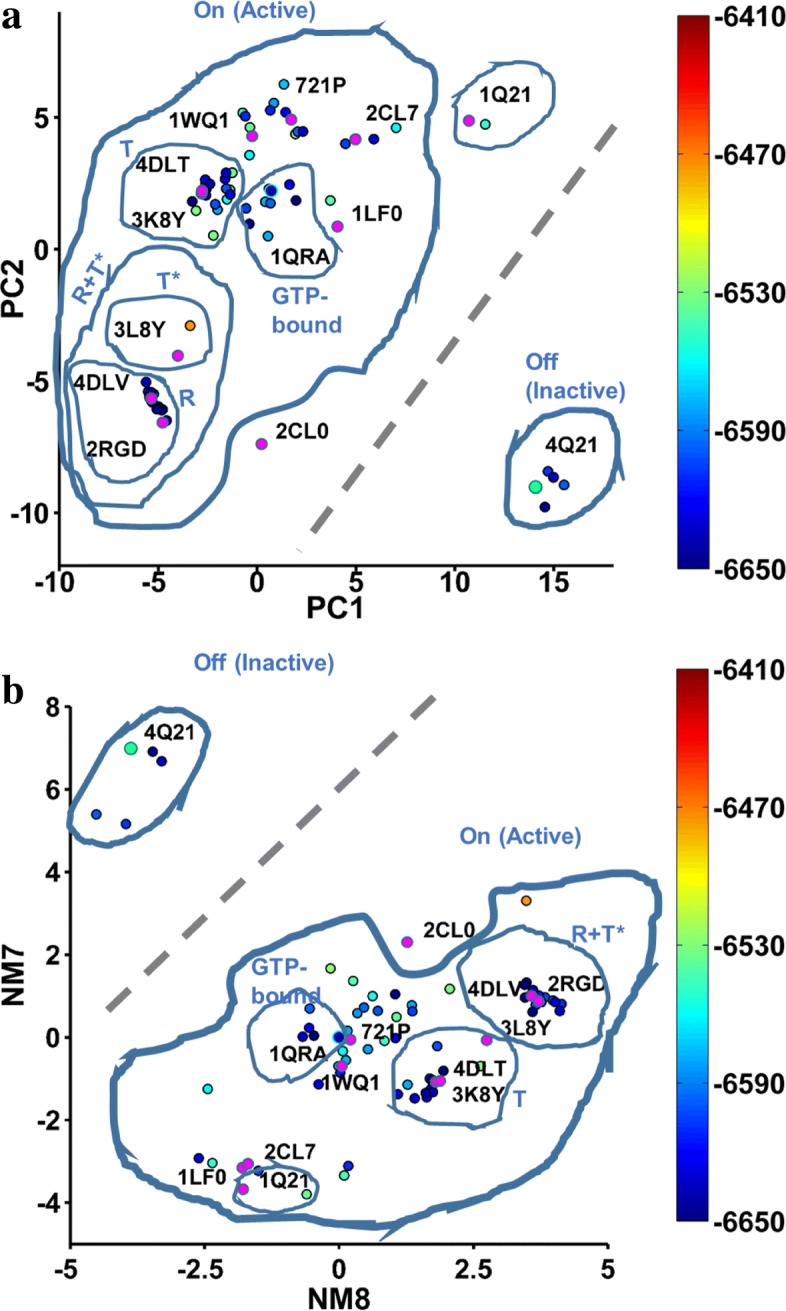
Comparison of Projections: WT-minimized, known structures of H-Ras are projected onto PC1 and PC2 in (**a**) and onto 1QRA-derived NM8 and NM7 in (**b**). The projections are colorcoded based on all-atom Amber ff14SB energies. PDB ids are shown alongside projections of selected structures. Annotations indicate known states and substates relevant for function

In Fig. 6a-b we compare the NMs to PCs via the organization they induce of experimentally-known structures of a protein. Specifically, Fig. 6a shows projections of crystallographic structures of H-Ras (stripped to CA atoms) on the top two PCs, PC1 and PC2. Figure 6b shows projections of these structures on 1QRA-derived NM8 and NM7 (similar results are obtained using the 4Q21-derived slowest NMs). Figure 6 shows that the NMs also encode the organization of the underlying, unknown energy landscape. The annotations in Fig. 6a-b synthesize wet- and dry-laboratory knowledge on H-Ras states and substates.

Altogether, Fig. 6 shows that the NM-based projections preserve the separation of the On and Off states, together with the co-localization of known structures corresponding to the T (tardy) versus the R+T* (reactive and hydrolyzed tardy) substates. Deformations are present; e.g., the R and T* states are not separable by NM8 and NM7, and smaller substates are also penetrated by projections of structures of other substates. These results support the premise that the NMs can serve as variable axes along which to “fill in” the unknown energy landscape. Based on the constraint to keep the dimensionality low, the rest of the analysis is on structures obtained from SoPriM-NMA with the top ten (m = 10) NMs as variable axes.

### Comparison of ensembles generated with SoPriM-PCA and SoPriM-NMA

**Fig. 7:**
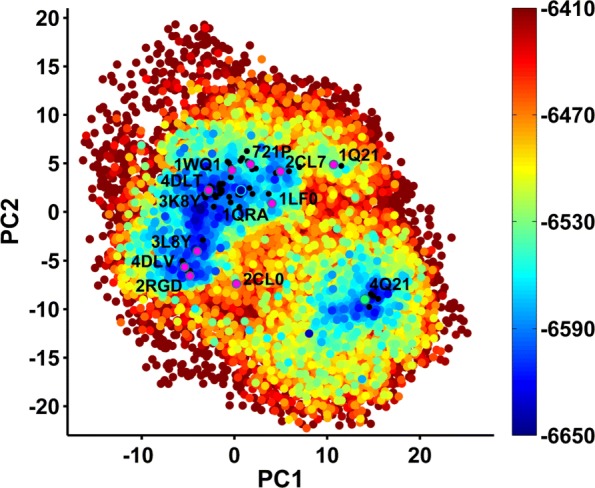
Landscape Probed with SoPriM-PCA: Structures obtained with SoPriM-PCA are projected onto PC1 and PC2. The projections are color-coded based on Amber ff14SB energies. Projections of known structures are also shown, and selected ones are annotated with their PDB ids to allow visualization of the known functional states

**Fig. 8:**
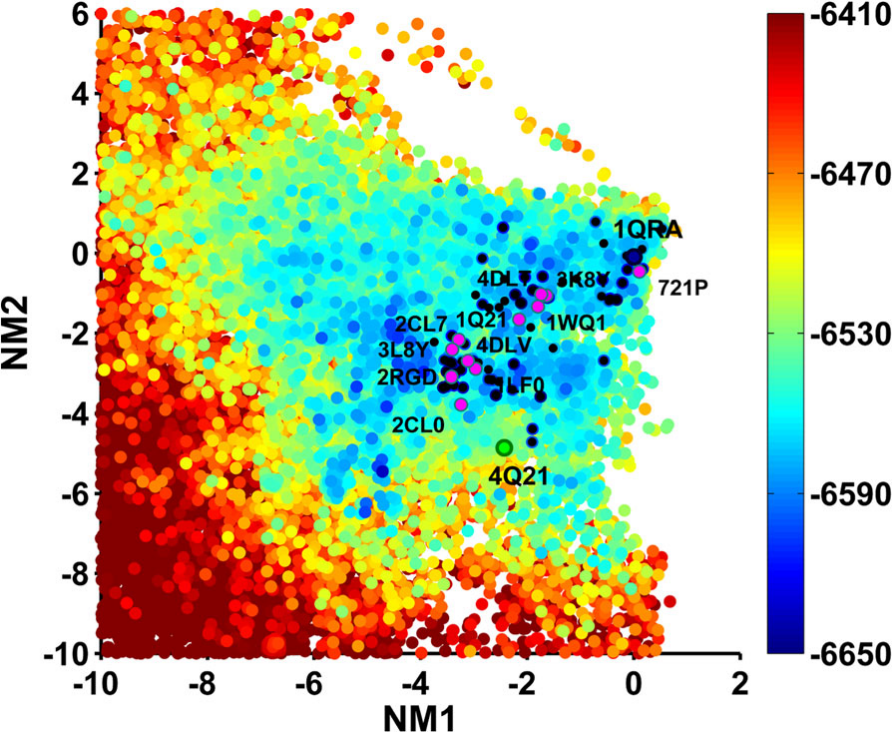
Landscape Probed with SoPriM-NMA (on NM1 and NM2): Structures obtained with SoPriM-NMA are projected onto NM1 and NM2. The projections are color-coded based on Amber ff14SB energies. Projections of known structures are also shown, and selected ones are annotated with their PDB ids to allow visualization of the known functional states

**Fig. 9:**
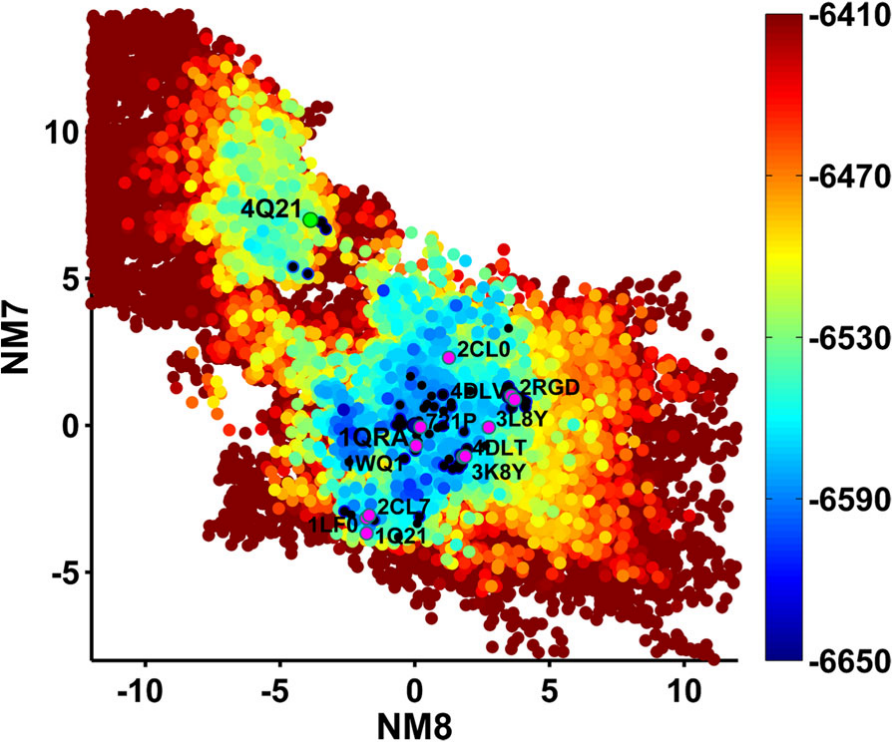
Landscape Probed with SoPriM-NMA (on NM8 and NM7): Structures obtained with SoPriM-NMA are projected onto NM8 and NM7. The projections are color-coded based on Amber ff14SB energies. Projections of known structures are also shown, and selected ones are annotated with their PDB ids to allow visualization of the known functional states

Below we relate results obtained when using the 1QRA-derived NMs but seeding the initial population of structures with all known PDB structures (threaded onto the WT and Amber ff14SB minimized); many other settings are analyzed but not shown here in the interest of space (such as using only the structure from which NMs are derived in the initial population, using 4Q21-derived NMs, etc.). Figures 7, 8 and 9 show the computed 2D energy landscape by drawing 2D projections of computed structures onto the top two axes and color-coding the projections by the Amber ff14SB energies of the corresponding structures. Figure 7 shows the PC1-PC2 landscape and serves as the baseline, showing the ability of SoPriM-PCA to reproduce the main On and Off states and even substates probed in the wet laboratory (as related in prior work). Figures 8 and 9 show the NM1-NM2 and NM8-NM7 landscapes, respectively, obtained when projecting SoPriM-NMA computed structures. The smaller substates are not as well populated as when using the top ten PCs as variables. However, the main On and Off states are captured well, particularly in Fig. 9, which separates these states, reproducing the presence of an energy barrier also reported in other studies based on Molecular Dynamics [23].

Better results are obtained when using the 4Q21-derived NMs (data not shown here). When the initial population is seeded to contain only one structure, the exploration capability of SoPriM-NMA suffers (data not shown), as more time is needed to expand to other regions of the structure space.

## Discussion

This study shows that much information can be inferred on the slow dynamics and the energy landscape even when only one structure is available for a protein under investigation. The SoPriM framework allows leveraging the normal modes extracted off a structure via normal mode analysis to build a sample-based representation of the underlying energy landscape that reveals functional states and substates and separating barriers. The presented results show that there is sufficient information encoded in the top ten normal modes about the equilibrium dynamics of a protein. Deformations of the landscape are present and cannot be corrected with information on only one structure. As demonstrated, the major states of a protein can be recovered, but not all substates can be separated. The presented work has highlighted that separability of functional states may only emerge on specific pairs of normal modes. While these modes are expected to be among the top few when extracted off a “closed” structure, they may not be so when extracted off an “open” structure. However, even a landscape reconstructed with informaton extracted off one structure can provide precious insight for function and function modulation. Moreover, the landscape may provide guidance on which regions of the structure space need to be further characterized in the wet laboratory.

## Conclusion

While the availability of more wet-laboratory structural data is desired for a protein under investigation, the study presented here opens further lines of enquiry onto how to reconstruct energy landscapes in the absence of such data. For instance, in the absence of any experimentally-known structural model on a protein of interest, structures of members in a protein’s superfamily can be leveraged. Issues regarding differences in lengths among members, particularly in gaps resulting from multiple sequence alignment, have to be resolved so that modes of motion can be defined for the protein of interest. Leveraging members of a protein’s superfamily constitutes a promising direction that we will investigate in future work.

## References

[1] Boehr DD, Nussinov R, Wright PE (2009). The role of dynamic conformational ensembles in biomolecular recognition. Nat Chem Biol.

[2] Nussinov R, Wolynes PG (2014). A second molecular biology revolution? The energy landscapes of biomolecular function. Phys Chem Chem Phys.

[3] Maximova T, Moffatt R, Ma B, Nussinov R, Shehu A (2016). Principles and Overview of Sampling Methods for Modeling Macromolecular Structure and Dynamics. PLoS Comp Biol.

[4] Russel D, Lasker K, Phillips J, Schneidman-Duhovny D, Veláquez-Muriel JA, Sali A (2009). The structural dynamics of macromolecular processes. Curr Opin Cell Biol.

[5] Shehu A. Probabilistic Search and Optimization for Protein Energy Landscapes In: Aluru S, Singh A, editors. Handbook of Computational Molecular Biology. Chapman & Hall/CRC: 2013.

[6] Shehu A, Plaku E (2016). A Survey of omputational Treatments of Biomolecules by Robotics-inspired Methods Modeling Equilibrium Structure and Dynamics. J Artif Intel Res.

[7] Maximova Tatiana, Plaku Erion, Shehu Amarda (2018). Structure-Guided Protein Transition Modeling with a Probabilistic Roadmap Algorithm. IEEE/ACM Transactions on Computational Biology and Bioinformatics.

[8] Sapin Emmanuel, De Jong Kenneth A., Shehu Amarda (2018). From Optimization to Mapping: An Evolutionary Algorithm for Protein Energy Landscapes. IEEE/ACM Transactions on Computational Biology and Bioinformatics.

[9] Sapin E, Carr DB, De Jong KA, Shehu A (2016). Computing energy landscape maps and structural excursions of proteins. BMC Genomics.

[10] Maximova T, Carr D, Plaku E, Shehu A (2016). Sample-based Models of Protein Structural Transitions. ACM Conf Bioinf & Comp Biol (BCB).

[11] Maximova T, Plaku E, Shehu A (2015). Computing Transition Paths in Multiple-Basin Proteins with a Probabilistic Roadmap Algorithm Guided by Structure Data. IEEE Intl. Conf. Bioinf. & Biomed.

[12] Clausen R, Shehu A (2015). A Data-driven Evolutionary Algorithm for Mapping Multi-basin Protein Energy Landscapes. J Comp Biol.

[13] Clausen R, Ma B, Nussinov R, Shehu A (2015). Mapping the Conformation Space of Wildtype and Mutant H-Ras with a Memetic, Cellular, and Multiscale Evolutionary Algorithm. PLoS Comput Biol.

[14] Qiao W, Maximova T, Plaku E, Shehu A (2017). Statistical Analysis of Computed Energy Landscapes to Understand Dysfunction in Pathogenic Protein Variants. ACM Conf on Bioinf and Comput Biol Workshops (BCBW): Comput Struct Biol Workshop (CSBW).

[15] Tirion MM (1996). Large amplitude elastic motions in proteins from a single parameter, atomic analysis. Phys Rev Lett.

[16] Bahar I, Lezon TR, Yang LW, Eyal E (2010). Global dynamics of proteins: bridging between structure and function. Annu Rev Biophys.

[17] Das A, Gur M, Cheng MH, Jo S, Bahar I, Roux B (2014). Exploring the Conformational Transitions of Biomolecular Systems Using a Simple Two-State Anisotropic Network Model. PLoS Comput Biol.

[18] Al-Bluwi I, Vaisset M, Siméon T, Cortés J (2013). Modeling protein conformational transitions by a combination of coarse-grained normal mode analysis and robotics-inspired methods. BMC Struct Biol.

[19] Schuyler AD, Jernigan RL, Wasba PK, Ramakrishnan B, Chirikjian GS (2009). Iterative cluster-NMA: a tool for generating conformational transitions in proteins. Proteins Struct Funct Bioinf.

[20] Kantarci-Carsibasi N, Haliloglu T, Doruker P (2008). Conformational transition pathways explored by Monte Carlo simulation integrated with collective modes. Biophys J.

[21] Grant BJ, Rodrigues AP, ElSawy KM, McCammon JA, Caves LS (2006). Bio3D: an R package for the comparative analysis of protein structures. Bioinformatics.

[22] McLachlan AD (1972). A mathematical procedure for superimposing atomic coordinates of proteins. Acta Crystallogr A.

[23] Grant BJ, Gorfe AA, McCammon JA (2009). Ras Conformational Switching: Simulating Nucleotide-Dependent Conformational Transitions with Accelerated Molecular Dynamics. PLoS Comput Biol.

